# Functional Characterization and Genomic Analysis of the Chlorantraniliprole-Degrading Strain *Pseudomonas* Sp. GW13

**DOI:** 10.3390/bioengineering6040106

**Published:** 2019-11-20

**Authors:** Wa Gao, Dongyang Li, Hong You

**Affiliations:** 1 Hubei Provincial Cooperative Innovation Center of Industrial Fermentation, Key Laboratory of Fermentation Engineering (Ministry of Education), School of Food and Biological Engineering, Hubei University of Technology, Wuhan 430068, China; wgao@mail.hbut.edu.cn; 2 State Key Laboratory of Cotton Biology, Institute of Cotton Research, Chinese Academy of Agricultural Sciences, Anyang, Henan 455000, China; hzaulidongyang@163.com; 3 Hubei Insect Resources Utilization and Sustainable Pest Management Key Laboratory, College of Plant Science and Technology, Huazhong Agricultural University, Wuhan 430070, China

**Keywords:** chlorantraniliprole, co-metabolism, *Pseudomonas*, draft genome

## Abstract

Chlorantraniliprole (CAP) is a widely used insecticide in many areas due to its excellent insecticidal ability and mammalian safety, however, the removal of CAP has not been extensively studied. In this study, a bacterial strain GW13, which is capable of co-metabolizing CAP, was isolated from a vegetable field soil. The strain was identified as *Pseudomonas* sp. based on its physico-biochemical characteristics and 16S rRNA gene analysis. The bacterial strain GW13 could degrade CAP through co-metabolism, and glucose was the best additional carbon resource. In the presence of 1.0 g/L glucose, GW13 could co-metabolize over 80% of 200 mg/L CAP in 24 h. The degradation rate increased after 6 h and slowed again after 10 h. The GW13 genome analysis revealed many genes associated with metabolism, showing the degradation mechanism of GW13 from the genomic perspective. The EAWAG-BBD (Swiss Federal Institute of Aquatic Science and Technology Biocatalysis/Biodegradation Database) prediction results showed that the main pathway for CAP degradation is amide hydrolysis, which is consistent with many genes associated with amidase in the GW13 genome. This study may facilitate research on CAP biodegradation mechanisms in the environment.

## 1. Introduction

Chlorantraniliprole (CAP), an insecticide of the anthranilic diamide chemical group was developed by DuPont [1]. CAP acts on insects by activating ryanodine receptors, which are specific and effective [2]. Because of the unique action mode and mammalian safety of CAP, it is widely used to control chewing pest insects, such as Lepidoptera, Diptera and Coleoptera insects, and CAP plays an important role in integrated pest management programs [3,4]. While many studies have focused on CAP application, few studies have investigated the degradation mechanism of CAP in the environment [5].

With the increasing service time of CAP, the accumulation of residues in soil and the environmental risks need to be considered, even though CAP exhibits minimal mammalian toxicity [6]. In consideration of public health and ecological environmental protection, some countries or regulatory bodies, such as China, the European Union, and the Codex Alimentarius Commission, have made standards or laws to restrict the residue levels of CAP in food. Because of the limited biodegradation of CAP, photolysis and hydrolysis are the two primary methods of CAP degradation in the environment. In previous studies, CAP was hydrolyzed with a half-life of approximately 10 days at pH 9, and the photolytic half-life of CAP in sterile aqueous buffer solution (pH 7.0) under continuous irradiation was 0.37 days [7,8,9].

Microorganisms play an important role in the removal of complex compounds [10], and they are a highly efficient, safe, and cost-effective way to remediate contaminated sites [11]. However, few studies have been reported on the biodegradation of CAP by bacterial strains. To date, only one strain belonging to *Pseudomonas* has been isolated from an agricultural field, and this strain can also degrade imidacloprid, endosulfan and coragen [12]. Among the biodegradation processes, co-metabolism is an important means of biodegrading environmental pollutants; it has been defined as the transformation of a nongrowth substrate in the obligatory presence of a growth substrate or another biodegradable compound, and a good example of co-metabolism is the biodegradation of emerging trace organic contaminants (EOCs) [13].

In this study, we obtained a bacteria strain GW13 capable of degrading CAP from a vegetable field soil by an enrichment technique. The isolation, identification, and characterization of GW13 was studied. In addition, the draft genome of GW13 was sequenced and analyzed. This work not only helped to characterize the CAP-degrading bacteria but also enhanced the understanding of the CAP biodegradation mechanism in the environment. These results may be valuable for research on CAP biodegradation and co-metabolism.

## 2. Materials and Methods

### 2.1. Chemicals and Media

Chlorantraniliprole (CAP), purity 98%, was purchased from DuPont (Shanghai, China). The stock solution of CAP was prepared in dimethylsulfoxide (DMSO). Chromatography-grade reagents were purchased from Sigma-Aldrich (St. Louis, USA). All other chemicals and solvents were of analytical grade. Molecular biology reagents were purchased from Sinopharm Chemical Reagent (Shanghai, China). Luria-Bertani (LB) liquid medium consisted of (per liter of distilled water) 10 g of tryptone, 5.0 g of yeast extract, and 5.0 g of sodium chloride. The pH was adjusted to pH 7.0. The solid medium contained 2.0% agar. Mineral salt medium (MM) contains (per L) 1.5 g of NH_4_NO_3_, 0.5 g of KH_2_PO_4_, 1.5 g of K_2_HPO_4_·3H_2_O, 0.2 g of MgSO_4_, and 0.5 g of NaCl. The pH was adjusted to pH 7.0. The concentration of CAP added to the MM was based on the requirement of each experiment.

### 2.2. Screening and Isolation of CAP-Degrading Strain

The soil was obtained from a vegetable field in a southern suburb of Wuhan, China. To enrich the CAP-degrading strains, the sample was cultured in 250 mL bottles containing 100 mL MM with CAP (50 mg/L) and glucose (1 g/L) as the source of carbon and energy, and the dissolution of CAP was promoted by DMSO. The bottle was incubated in a shaking air bath at 30 °C and 220 rpm in the dark. After incubation for 7–10 days, 5 mL of the enrichment culture was transferred into 100 mL of fresh MM containing CAP (50 mg/L) and glucose (1 g/L). The rate of CAP removal was determined by HPLC. The concentration of CAP in MM increased gradually when the rate of CAP removal was greater than 50%.

The enrichment that was able to degrade CAP was serially diluted and then spread onto LB agar plates containing 50 mg/L CAP. Then, a number of bacterial strains were isolated and purified. These strains were incubated in 50 mL bottles containing 10 mL MM with CAP (50 mg/L) and glucose (1 g/L) for 7 days. The CAP removal ability of these strains was tested by HPLC. One strain named GW13 was selected for further studies because it had the highest CAP removal ability.

### 2.3. Identification of CAP-Degrading Strain GW13

The physiological features of strain GW13 were tested by Vitek^®^ 2 compact (bioMerieux, Craponne, France). Genomic DNA of GW13 was extracted using an E.Z.N.A.^®^ Bacterial DNA Kit (Omega Bio-tek, Norcross, GA, USA). Universal primers 27F (5′-AGAGTTTGATCCTGGCTCAG-3′) and 1492R (5′-GGTTACCTTGTTACGACTT-3′) were used for PCR amplification of the 16S rRNA genes. The PCR products were cloned into the pGEM-T Easy vector (Promega, USA) and transformed into *Escherichia coli* DH5α cells. The clones were sequenced by Tsingke Biological Technology Co. Ltd. (Beijing, China). The obtained sequences were subjected to an EzBioCloud homology search [14]. Multiple sequence alignments were conducted with the selection of high homology sequences using Clustal W [15]. The phylogenetic tree was constructed using the neighbor-joining method by MEGA 7.0 version [16].

### 2.4. Biodegradation of CAP of Strain GW13

Strain GW13 was incubated in LB media at 30 °C and 220 rpm until the OD600 of the culture solution reached 2.0. Then, the strain was aseptically collected (6000 rpm, 5 min), washed twice with PBS solution, and resuspended in an equal volume of PBS solution. To test the degradation rate of GW13, 2% of resting cells (OD600 = 2.0) was added to 20 mL MM containing CAP (50 mg/L) and glucose (1 g/L). It was incubated at 30 °C and 220 rpm. The non-inoculated culture served as a control. The residual concentration was measured by HPLC. All experiments were performed in triplicate. The data are represented as the mean ± standard deviation(SD) for triplicate incubations. Statistical analyses (one-way analysis of variance, ANOVA, and Dunnett’s test) were conducted using SPSS 25.0 for windows (SPSS, Armonk, NY, USA: IBM Corp).

### 2.5. Chemical Analysis

The substrate residues in the cultures were extracted by ethyl acetate, and then the solution was dried and re-dissolved by methyl alcohol. The extracting solution was analyzed on an Agilent 1260 HPLC system equipped with a C18 reversed-phase column (250 mm × 4.60 mm, 5 μm) with UV detection at 265 nm based on retention time and the peak area of the pure standard. A mixture of methanol and water (80:20, *v*/*v*) was used as the mobile phase at a low rate of 1.0 mL/min, and the column temperature was 30 °C.

### 2.6. Genome Sequencing, Assembly, and Annotation

Genomic DNA was extracted with a DNA isolation kit (Omega Bio-tek, Norcross, GA, USA). Whole-genome sequencing was performed on the Illumina HiSeq 2500-PE125 platform with MPS (massively parallel sequencing) Illumina technology at the Beijing Novogene Bioinformatics Technology Co., Ltd. Then, all good quality paired reads were assembled using SOAPdenovo [17,18]. The Kyoto Encyclopedia of Genes and Genomes (KEGG) database [19,20] and Clusters of Orthologous Groups (COG) database [21] were used to predict the functions.

## 3. Results

### 3.1. Isolation and Characterization of the CAP-Degrading Strain

**Figure 1:**
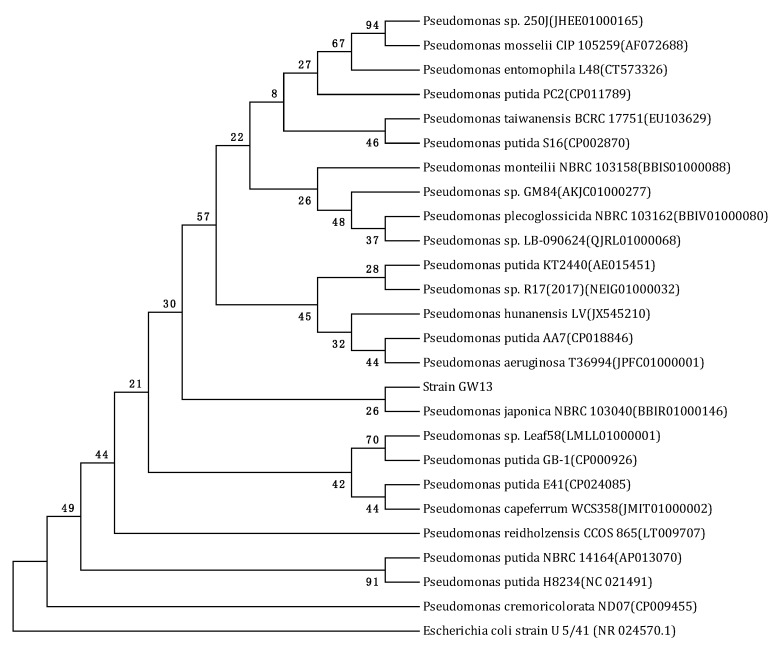
Phylogenetic tree derived from the 16S rRNA gene sequence of strain GW13 and sequences of related species in EzBioCloud by the neighbor joining method. Bootstrap values (%) are indicated at the nodes (based on 1000 samplings). The accession number for each microorganism is shown in parentheses after the species name. *Escherichia coli* strain U5/41 (NR 024570.1) was used as the out-group.

Several bacterial strains were isolated from the vegetable field soil sample after enrichment in a CAP-containing medium for approximately three months. Strain GW13 was chosen for further study. Strain GW13 was an aerobic, Gram-negative, short-rod-shaped bacterium. Colonies on LB agar plates were smooth, opaque, and yellowish-white in color. The phylogenetic tree showed strain GW13 is closest to *P. japonica* NBRC 103040. The Vitek analysis also confirmed that this strain belongs to the genus *Pseudomonas* (Table 1). Based on the morphological characteristics, physico-biochemical characteristics, and 16S rRNA gene analysis (Figure 1), strain GW13 was identified as *Pseudomonas* sp.

### 3.2. Utilization of CAP for Growth by Strain GW13

**Figure 2:**
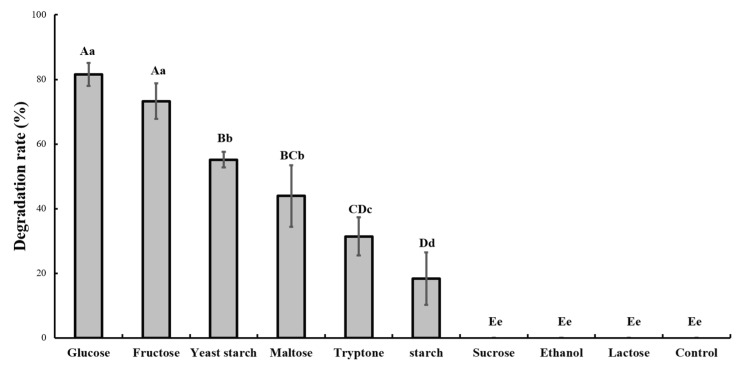
Effect of different carbon sources (1 g/L) on the degradation of 50 mg/L chlorantraniliprole (CAP) by strain GW13. The degrading amount of CAP was measured after incubation in 25 °C and pH 7 for 24 h. The data are represented as the mean ± standard deviation (SD) for triplicate incubations. Different letters above the bars indicate significant differences among different treatments at *p* < 0.05 (uppercase) or *p* < 0.01 (lowercase) (one-way analysis of variance, ANOVA, and Dunnett’s test).

In MM medium supplemented with glucose, strain GW13 degraded over 80% of 200 mg/L CAP in one day. However, strain GW13 cannot degrade CAP in MM medium without other carbon resources. These results showed that the other carbon resources greatly affected the degradation of CAP by strain GW13. When strain GW13 grew with glucose as the carbon source, it simultaneously co-metabolized CAP. Among several common carbon sources, glucose, fructose and yeast starch also had a robust promoting effect, and maltose, tryptone, starch had a weak promoting effect, but sucrose, ethanol, and lactose had little promoting effect (Figure 2).

**Figure 3:**
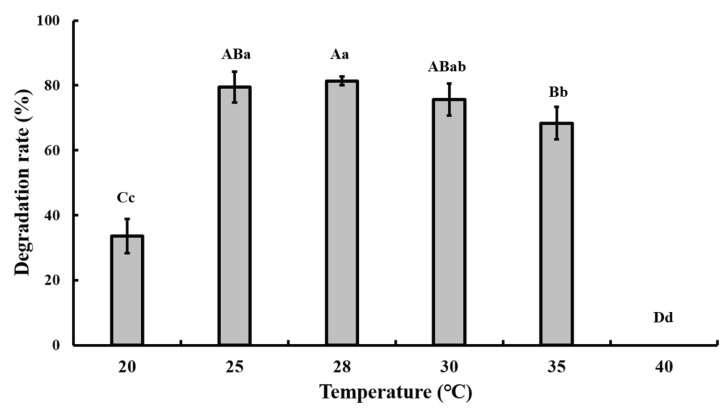
Effect of different temperatures on the degradation of 50 mg/L CAP by strain GW13. The degrading amount of CAP was measured after incubation in pH 7 and 1 g/L glucose adjunction for 24 h. The data are represented as the mean ± standard deviation (SD) for triplicate incubations. Different letters above the bars indicate significant differences among different treatments at *p* < 0.05 (uppercase) or *p* < 0.01 (lowercase) (one-way analysis of variance, ANOVA, and Dunnett’s test).

**Figure 4:**
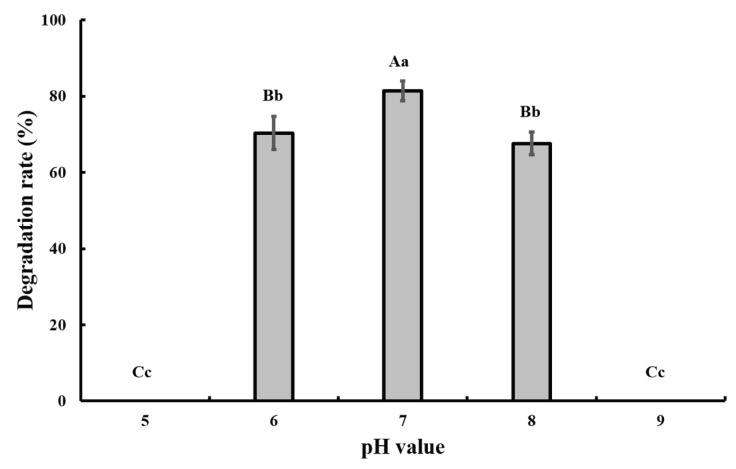
Effect of different pH values on the degradation of 50 mg/L CAP by strain GW13. The degrading amount of CAP was measured after incubation in 28 °C and 1 g/L glucose adjunction for 24 h. The data are represented as the mean ± standard deviation (SD) for triplicate incubations. Different letters above the bars indicate significant differences among different treatments at *p* < 0.05 (uppercase) or *p* < 0.01 (lowercase) (one-way analysis of variance, ANOVA, and Dunnett’s test).

The optimal pH and temperature for CAP degradation were tested in the batch experiment, and the results showed that pH 7.0 and 28 °C were the optimal conditions (Figure 3 and Figure 4).

**Figure 5:**
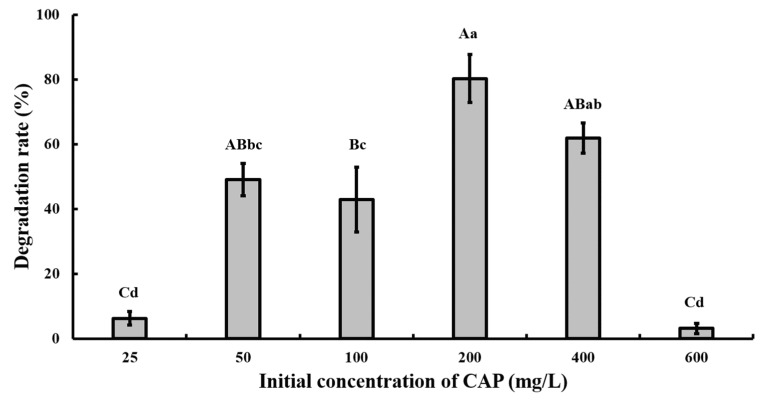
Effect of different initial CAP concentrations on the degradation of CAP by strain GW13. The degrading amount of CAP was measured after incubation in 28 °C, pH 7 and 1 g/L glucose adjunction for 24 h. The data are represented as the mean ± standard deviation (SD) for triplicate incubations. Different letters above the bars indicate significant differences among different treatments at *p* < 0.05 (uppercase) or *p* < 0.01 (lowercase) (one-way analysis of variance, ANOVA, and Dunnett’s test).

**Figure 6:**
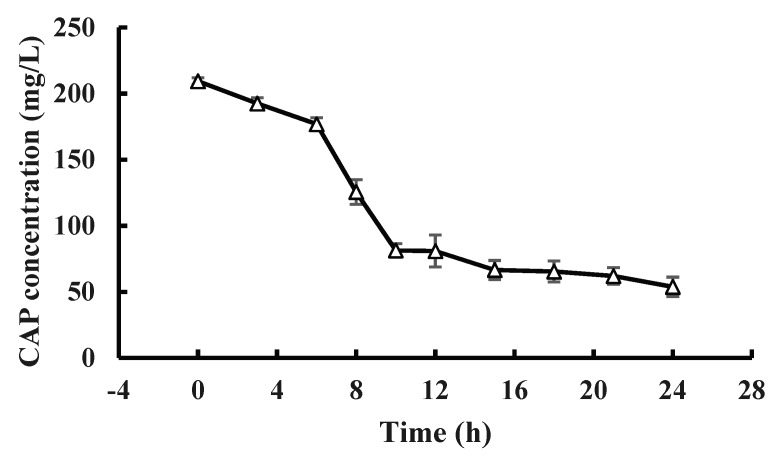
CAP (200 mg/L) degradation curve of GW13 in 24 h. The degrading amount of CAP was measured after incubation in 28 °C, pH 7 and 1 g/L glucose adjunction for 24 h. The data are represented as the mean ± standard deviation (SD) for triplicate incubations.

At pH 7.0 and 28 °C, strain GW13 degraded CAP at a wide range of concentrations (from 50 mg/L to ~400 mg/L), and strain GW13 had the best degradation efficiency when the initial concentration of CAP was 200 mg/L (Figure 5). Under optimal growth conditions, the degradation curves of CAP by strain GW13 at 200 mg/L CAP initial concentration are shown in Figure 6. The rate of degradation increased after 6 h and slowed again after 10 h.

### 3.3. Overview of the Pseudomonas Sp. Strain GW13 Genome

**Figure 7:**
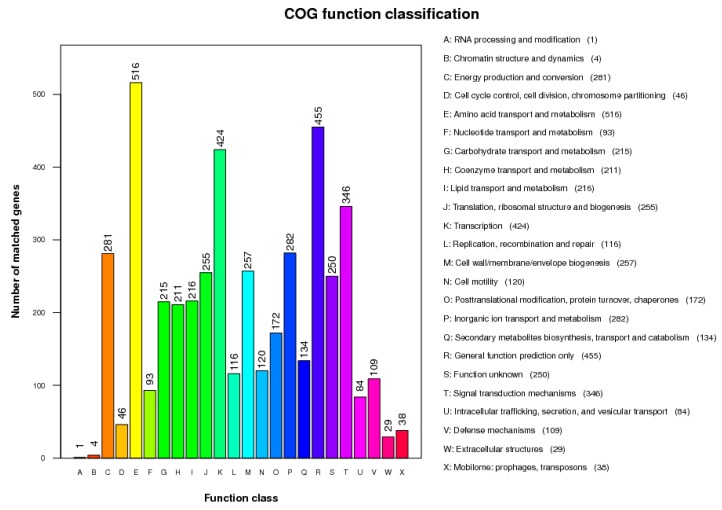
Clusters of Orthologous Groups (COG) database function classification of strain GW13.

**Figure 8:**
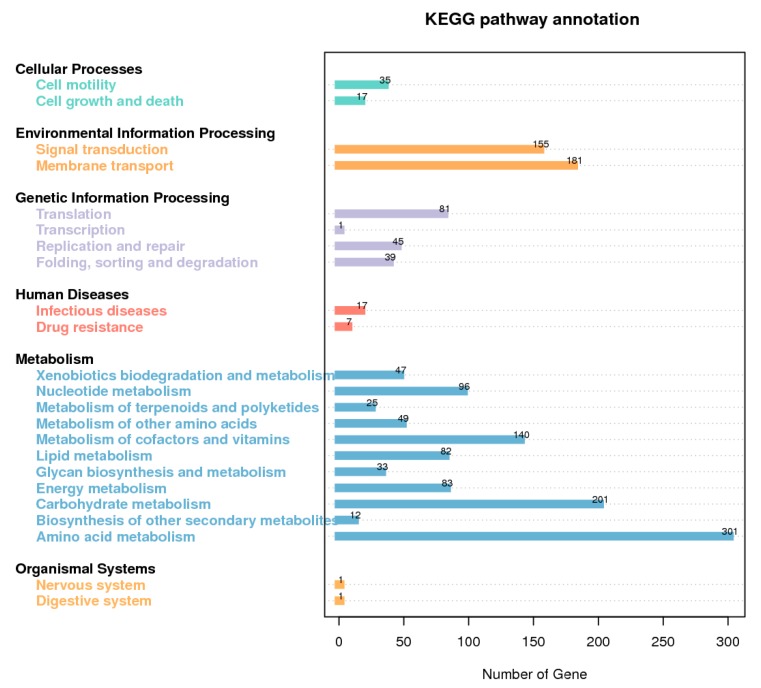
Kyoto Encyclopedia of Genes and Genomes (KEGG) database metabolic pathway classification map of GW13 genome.

The total length of the draft genome of strain GW13 was 5,687,231 bp with a G + C content of 65.96%. The genome was assembled into 128 contigs using SOAPdenovo [17,18]. The chromosome contains seven copies of rRNA genes, 65 tRNA genes, and 5071 protein-coding genes, and 4019 protein-coding genes could be assigned to 24 categories of clusters of orthologous groups (COGs), amino acid transport and metabolism (516 genes), carbohydrate transport and metabolism (215 genes), coenzyme transport and metabolism (211 genes), lipid transport and metabolism (216 genes) clustered many genes, which showed good metabolic capability of GW13 (Figure 7). The gene functions of strain GW13 were predicted with the KEGG database (Figure 8). We obtained 52 genes for cellular processes, 336 genes for environmental information processing, 166 genes for genetic information processing, 24 genes for human diseases, 1069 genes for metabolism, and two genes for organism systems.

Average nucleotide identity (ANI) analysis revealed that *P. entomophila* L48 (89.06%), *P. putida* GB-1 (87.15%), *P. putida* NBRC 14164 (87.37%), *P. putida* S16 (87.66%), and *P. putida* KT2440 (87.15%) are all close to strain GW13 (Table 2).

**Figure 9:**
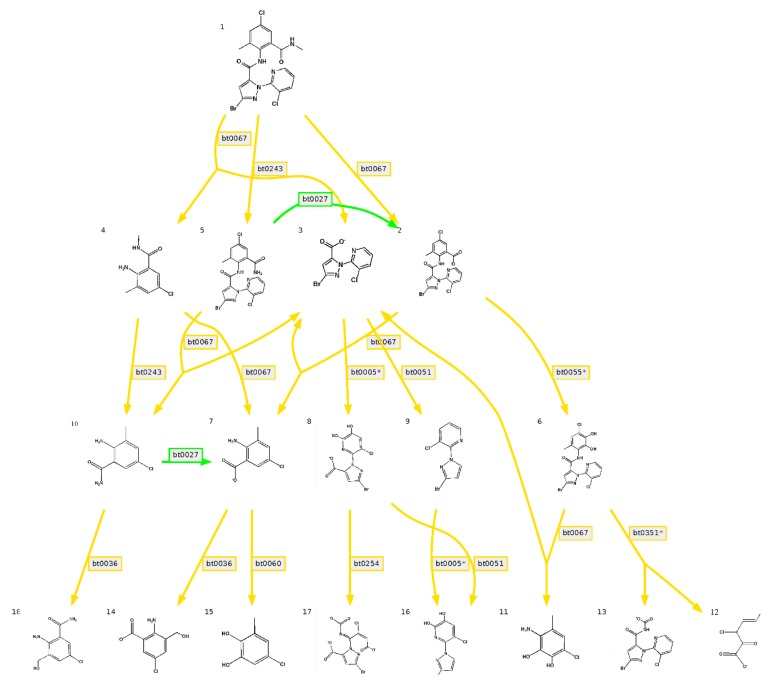
Predicted degradation pathway map of chlorantraniliprole by the Swiss Federal Institute of Aquatic Science and Technology Biocatalysis/Biodegradation Database (EAWAG-BBD) pathway prediction system, green lines indicate very likely reaction, yellow lines indicate likely reaction (a five-point Likert scoring scale, Very likely reaction/Likely reaction/Possible reaction (neutral)/Unlikely reaction/Very unlikely reaction, is used to indicate the aerobic likelihood of reaction rules in the EAWAG-BBD pathway prediction system). Bt0027 indicates a pathway of primary Amide -> Carboxylate in EAWAG-BBD pathway prediction system, bt0067 indicates a pathway of secondary Amide -> Carboxylate + primary Amine, bt0243 indicates a pathway of *N*-substituted Amide -> Amide + Aldehyde or Ketone, bt0005 indicates a pathway of vic-unsubstituted Aromatic -> vic-Dihydroxyaromatic, bt0051 indicates a pathway of 2- or 3-substituted Carboxylate -> RH + CO2, bt0055 indicates a pathway of 1-carboxy-2-unsubstituted Aromatic -> Catechol derivative, bt0036 indicates a pathway of aromatic Methyl -> primary Alcohol, bt0060 indicates a pathway of vic-Hydroxycarboxyaromatic -> Catechol derivative, bt0254 indicates a pathway of vic-Dihydroxyaromatic -> intradiol ring cleavage, bt0351 indicates a pathway of vic-Dihydroxybenzenoid -> 2-Oxopent-4-enoate derivative + Carboxylate.

We predicted the degradation of CAP using the EAWAG-BBD Pathway Prediction System [22]; the result is shown in Figure 9. Prediction cannot entirely represent the actual situation, but it is important to further research the degradation mechanism. Based on predictions, the main degrading pathway of CAP is amide hydrolysis by amidase. According to sequence analysis, there were 103 genes associated with amidase. In addition, there were also many genes associated with the degradation of xenobiotics, including 55 genes for benzoate degradation, 53 genes for butanoate metabolism, 44 genes for methane metabolism, 42 genes for glyoxylate and dicarboxylate metabolism, 42 genes for propanoate metabolism, 33 genes for aminobenzoate degradation, 32 genes for naphthalene degradation, 24 genes for bisphenol degradation, 11 genes for polycyclic aromatic hydrocarbon degradation, 10 genes for toluene degradation, 10 genes for styrene degradation, 24 genes for chloroalkane and chloroalkene degradation, and seven genes for chlorocyclohexane and chlorobenzene degradation.

## 4. Discussion

Chlorantraniliprole (CAP) is a widely used insecticide, and concern for the environmental fate of this insecticide is needed because of the potential risks of CAP residues in soil and the environment. The US Environmental Protection Agency (USEPA) has paid considerable attention to the removal of CAP. The main degradation methods are photolysis, hydrolysis and biodegradation in the liver of some animals [6]. However, CAP-degrading microorganisms are rarely reported. To date, the only published report is from Gupta et al., who isolated a bacterial strain identified as *Pseudomonas* sp. RPT 52 that is capable of degrading imidacloprid, endosulfan and coragen [12].

In this study, a CAP-degrading bacterial strain, GW13, was isolated and identified as *Pseudomonas* sp. on the basis of the morphological characteristics, physico-biochemical characteristics and 16S rRNA gene analysis. In a previous study, the genus *Pseudomonas* is a valuable resource for degrading pesticides in the environment, such as ethametsulfuron-methyl, imazethapyr, buprofezin, chlorothalonil, o-nitriobenzaldehyde, and phenol [23,24,25,26,27,28]. Additionally, strain RPT 52, which is capable of degrading imidacloprid, endosulfan and coragen, also belongs to the genus *Pseudomonas* [12]. This broad degradation spectrum makes the genus *Pseudomonas* a valuable microorganism for bioremediation.

The degradation of CAP by strain GW13 requires the presence of other carbon resources. This process is consistent with the description of co-metabolism [12]. Co-metabolism is a different means of biodegradation; it relies on the nonspecific enzymes/cofactors of microorganisms and requires additional energy. The degradation products may appear more toxic or need further degradation in other ways. In other words, co-metabolism is not beneficial to the microorganism itself [29].

For co-metabolism, the type and quantity of the additional carbon resource is an important influencing factor [30]. In our study, different carbon resources showed different effects on the degradation rate. Among these tested carbon sources, glucose had the best effect on the degradation of CAP by strain GW13. An additional carbon resource is the carbon resource and energy resource of the microorganism, which may influence co-metabolic degradation efficiency and even the formation of byproducts. Zhong et al. showed that strain PheB4 (*Sphingomonas* sp.) has a more rapid co-metabolic degradation rate of polycyclic aromatic hydrocarbons (PAHs) when peptone, beef extract, or glucose serve as the additional carbon resource [31].

In addition, due to the competition of the nongrowth substrate and the growth substrate on the key metabolic enzymes in the co-metabolism reaction, the ratio of the nongrowth substrate and the growth substrate may strongly affect co-metabolism [30]. In our study, the appropriate initial concentration was 1 g/L glucose and 200 mg/L CAP. Thus, an inappropriate ratio of CAP and glucose may be the cause of slower CAP degradation after the tenth hour in the degradation curve. For the maximum degradation rate, the reason why CAP cannot be completely degraded may be the toxic degradation products or inappropriate ratio of CAP and glucose.

Co-metabolism is a multifaceted process that may be influenced by many factors, such as temperature, pH, oxygen content, availability of chemicals to microorganisms, presence of growth substrates, and physicochemical properties, molecular structures, and toxicity of chemicals [29], and these factors may influence each other. Thus, the interaction between these factors and co-metabolism remains to be fully elucidated.

Genome analysis is a powerful tool for characterizing microorganisms. According to the analysis of the GW13 genome, we obtained some useful tips. We learned that strain GW13 belongs to strains of *Pseudomonas* from a genome perspective. The genes associated with metabolism are more abundant than others, which showed the degradation function of GW13. The EAWAG-BBD prediction results indicated that many genes associated with amidase may be associated with CAP degradation. In addition, genes for the degradation of butanoate, naphthalene, bisphenol, polycyclic aromatic hydrocarbon, toluene, and styrene may be related to the degradation of the benzene ring in CAP. In addition, GW13 also has the potential to metabolize halide.

## 5. Conclusions

A novel *Pseudomonas* sp. strain GW13 capable of co-metabolizing CAP was isolated from soil collected from a vegetable field. Glucose was the best carbon source for co-metabolizing CAP by strain GW13. In addition, many genes associated with CAP degradation were predicted by the EAWAG-BBD pathway prediction system, and genes related to amidase may be an important means of CAP degradation. The present study facilitates future research investigating degrading microbes and enhances the understanding of CAP biodegradation in the environment.

## Figures and Tables

**Table 1:** Vitek analysis results for strain GW13, ‘+’ indicates a positive reaction, and ‘−‘ indicates a negative reaction.

Index	Name	Reaction	Index	Name	Reaction
PyrA	Pyrrolidinyl arylamine	−	dTRE	d-trehalose	−
IARL	l-arabitol	−	CIT	Citrate	+
dCEL	d-cellobiose	−	MNT	Malonate	−
BGAL	Beta-galactosidase	−	5 KG	5-keto-glucoside	−
H2S	H2S production	−	ILATk	l-LACTA TE alkalinisation	+
BNAG	β-*N*-acetylglucosidase	−	AGLU	Alpha-glucose	−
AGLTp	Glutamine arylamine	−	SUCT	Succinate alkali	+
dGLU	d-glucose	+	NAGA	Beta-*N*-Acetyl-Galactosaminidase	−
GGT	Y-glutamine transferase	+	AGAL	Alphagalactosidase	−
OFF	Fermente glucose	−	PHOS	Phosphatase	−
BGLU	Beta-glucosidase	−	GlyA	Glycine arylamine	−
dMAL	d-maltose	−	ODC	Ornithine decarboxylase	−
dMAN	d-mannitol	−	LDC	Lysine decarboxylase	−
dMNE	d-mannose	−	IHISa	Histidine assimilation	−
BXYL	Beta-xylosidase	−	CMT	COURMARATE	−
BAlap	Beta-alanine arylamine pNA	+	BGUR	Beta-glucuronidase	−
ProA	l-valine arylamine	+	O129R	O/129 resistance	+
LIP	Esterase	−	GGAA	Glutamate-glycine-arginine arylamine	−
PLE	PALATINOSE	−	IMLTa	l-malate assimilation	−
TyrA	Tyrosine arylamine	+	ELLM	ELLMAN	−
URE	Urease	+	ILATa	l-lactate assimilation	−
dSOR	d-sorbitol	−			

**Table 2:** Genomic characteristics of GW13 and other reference strains in EzBioCloud.

Strain	Genome Size (bp)	CDSs	rRNA Genes	tRNA Genes	GC %	ANIm %	Aligned %
GW13	5,687,221	5071	7	67	63.9	-	-
*Pseudomonas entomophila* L48	5,888,780	5073	23	78	64.2	89.06	73.03
*Pseudomonas putida* GB-1	6,078,430	5446	23	74	61.9	87.15	62.95
*Pseudomonas putida* NBRC 14164	6,156,701	5436	23	75	62.3	87.37	62.08
*Pseudomonas putida* S16	5,984,790	5467	20	67	62.3	87.66	62.30
*Pseudomonas putida* KT2440	6,181,873	5583	23	74	61.5	87.15	59.13
